# Comparison of Normocalcemic vs Hypercalcemic Primary Hyperparathyroidism in a Hypercalciuric Renal Stone Population

**DOI:** 10.1210/clinem/dgae162

**Published:** 2024-03-18

**Authors:** Caroline Halimi, Clemence Bor, Remi Chieze, Camille Saint-Jacques, Sophie Périé, Isabelle Wagner, Jean-Noel Talbot, Françoise Montravers, Emmanuel Letavernier, David Buob, Michel Daudon, Vincent Frochot, Jean-Philippe Haymann

**Affiliations:** Service ORL et chirurgie cervico-faciale, Hôpital Bichat, Assistance Publique-Hôpitaux de Paris, 75018 Paris, France; INSERM, Unité mixte de Recherche 1155, Kidney Research Centre, AP-HP, Hôpital Tenon, Sorbonne Université, 75020 Paris, France; Service de Nephrologie, Hôpital Européen de Paris, 93300 Aubervilliers, France; Service d’Explorations Fonctionnelles, Hôpital Tenon, Assistance Publique-Hôpitaux de Paris, 75020 Paris, France; Service d’Explorations Fonctionnelles, Hôpital Tenon, Assistance Publique-Hôpitaux de Paris, 75020 Paris, France; Service ORL et chirurgie cervico-faciale, Centre Hospitalier Privé Ambroise Paré Hartmann, 92200 Neuilly sur Seine, France; Service ORL et chirurgie cervico-faciale, Hôpital Tenon, Assistance Publique-Hôpitaux de Paris, 75020 Paris, France; Service de médecine nucléaire, Hôpital Tenon, Assistance Publique-Hôpitaux de Paris, 75020 Paris, France; Institut National des Sciences et Techniques Nucléaires (INSTN), 91190 Saclay, France; Service de médecine nucléaire, Hôpital Tenon, Assistance Publique-Hôpitaux de Paris, 75020 Paris, France; INSERM, Unité mixte de Recherche 1155, Kidney Research Centre, AP-HP, Hôpital Tenon, Sorbonne Université, 75020 Paris, France; Service d’Explorations Fonctionnelles, Hôpital Tenon, Assistance Publique-Hôpitaux de Paris, 75020 Paris, France; INSERM, Unité mixte de Recherche 1155, Kidney Research Centre, AP-HP, Hôpital Tenon, Sorbonne Université, 75020 Paris, France; Service d’anatomo-pathologie, Hôpital Tenon, Assistance Publique-Hôpitaux de Paris, 75020 Paris, France; Service d’Explorations Fonctionnelles, Hôpital Tenon, Assistance Publique-Hôpitaux de Paris, 75020 Paris, France; INSERM, Unité mixte de Recherche 1155, Kidney Research Centre, AP-HP, Hôpital Tenon, Sorbonne Université, 75020 Paris, France; Service d’Explorations Fonctionnelles, Hôpital Tenon, Assistance Publique-Hôpitaux de Paris, 75020 Paris, France; INSERM, Unité mixte de Recherche 1155, Kidney Research Centre, AP-HP, Hôpital Tenon, Sorbonne Université, 75020 Paris, France; Service d’Explorations Fonctionnelles, Hôpital Tenon, Assistance Publique-Hôpitaux de Paris, 75020 Paris, France

**Keywords:** primary hyperparathyroidism, normocalcemic primary hyperparathyroidism, hypercalcemic primary hyperparathyroidism, hypercalciuria, CaSR

## Abstract

**Context:**

Primary hyperparathyroidism (PHPT) is commonly diagnosed in the setting of hypercalcemia, whereas normocalcemic primary hyperparathyroidism (NHPT) may be misdiagnosed.

**Objective:**

Our objective was to compare patients with hypercalcemic hyperparathyroidism (HPHPT) vs patients with NHPT hypercalciuric renal stones.

**Methods:**

We took advantage of a routine calcium load test performed in patients with hypercalciuric renal stones to assess retrospectively among patients with PHPT the prevalence and characteristics of NHPT and HPHPT under a calcium-restricted diet

**Results:**

Among 1671 patients with hypercalciuria, 91 patients had a final diagnosis of PHPT (postload ionized calcium [iCa] > 1.31 mmol/L and parathyroid hormone [PTH] > 30 pg/mL). Prevalence of NHPT is 40% of all PHPT; however, according to total serum calcium, 4/35 NHPT and 7/56 HPHPT cases would have been misclassified in the other group. Eighteen of 35 NHPT and 40/56 HPHPT cases underwent parathyroidectomy. No significant characteristics relating to parathyroid weight, stone composition, or bone remodeling biomarkers were detected between groups. Although iCa is higher in HPHPT in the fasting state and after calcium load, we found no difference for calcium diet, 24-hour calciuria, or calcitriol. Renal calcium excretion postload increased by 303% in NHPT but only 176% in HPHPT (*P* = .01) likely explained by a lesser PTH decrease (*P* = .02). However, a strong negative association (*P* < .0001) detected between pooled preload and postload iCa and PTH only in the NHPT group suggests a persistent efficient PTH-CaSR control within the parathyroid glands in this group.

**Conclusion:**

Our data show the relevance of dynamic tests to unmask NHPT in patients with hypercalciuric renal stones.

Primary hyperparathyroidism (PHPT) is one of the most common endocrine disorders with increasing prevalence in recent decades due to routine biological screening. The current curative treatment is parathyroidectomy according to guidelines (1), with beneficial results in the long-term on bones and kidneys targets. Asymptomatic forms are increasingly diagnosed and raise the issue of the therapeutic management. Moreover, normocalcemic forms (normocalcemic primary hyperparathyroidism, NHPT), currently defined by “a normal total and ionized serum calcium level without any other known etiology for a secondary elevation of parathyroid hormone (PTH),” have been recognized as a singular entity whose natural history and physiopathology remain poorly understood (2, 3). Indeed, some patients may present long-term asymptomatic normocalcemia altogether with hypercalciuria and renal stones while other patients experience only bone mineral disorders or develop hypercalcemia during follow-up (4). The diagnosis is flawed by a potential inaccuracy of the measurements of total and ionized serum calcium and serum PTH level. Indeed, a normal ionized calcium serum level in the setting of an increased serum PTH concentration may lead to a diagnosis of NHPT if hypercalcemia is unmasked after a calcium load together with an inadequate serum PTH level inhibition (5). Alternatively, the persistence after a calcium load test of normal ionized calcium and PTH serum concentration rules out PHPT and drives one to a diagnosis of secondary hyperparathyroidism related to a normal/low ionized calcium serum level in a fasting state.

We took advantage of a routine calcium load test at our center (6) that was carried out in patients with hypercalciuric renal stones to retrospectively compare our populations of normocalcemic vs hypercalcemic PHPT (NHPT vs HPHPT, respectively) to get a better understanding of NHPT pathophysiology.

Indeed, when selecting a stringent criterion for NHPT based upon normal ionized calcium and not total calcium under a 2-day restricted diet and the onset of true hypercalcemia following a calcium load test with inappropriate PTH, our data pinpoint differences between NHPT and HPHPT in calcium homeostasis mainly related to better PTH inhibition and renal calcium excretion with no difference for intestinal calcium absorption or calcitriol regulation. Moreover, among patients who underwent surgery, despite a trend, no significant parathyroidectomy gland weight difference was detected between the 2 groups.

## Materials and Methods

### Population

**Figure 1.**
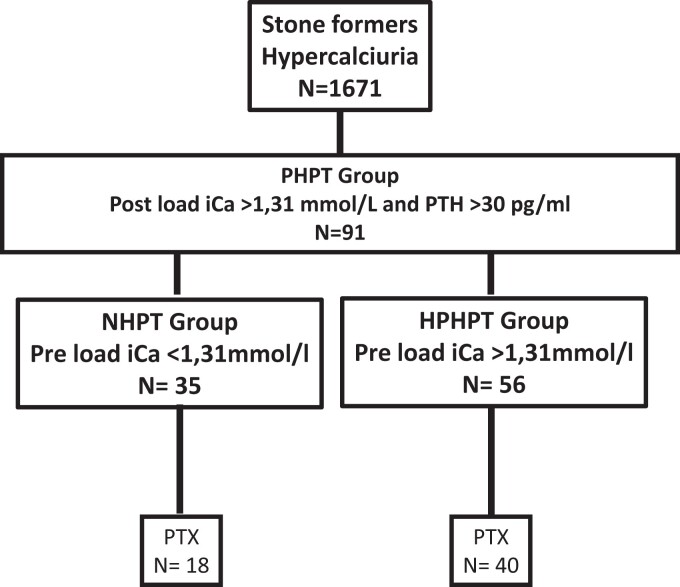
Flow chart. Among 1671 patients with hypercalciuria and renal stones referred to our department for a calcium load test over a 10 years. PHPT, primary hyperparathyroidism; NHPT, normocalcemic primary hyperparathyroidism; HPHPT, hypercalcemic primary hyperparathyroidism; PTX, parathyroidectomy.

From an initial cohort of 1671 patients with hypercalciuria and renal stones (Fig. 1) referred to our center in Tenon Hospital for a calcium load test, 91 patients with a final diagnosis of hyperparathyroidism were retrospectively included based upon the following criteria: the presence of a high serum ionized calcium level (>1.31 mmol/L) after a calcium load test associated with an inadequate PTH serum level (ie, >30 pg/mL).

Of notice, among our selection no patients with PHPT had an estimated glomerular filtration rate <60 mL/min/1.73 m^2^ or received medication interacting with bone metabolism (bisphosphonate, lithium, loop diuretic or thiazide, corticosteroid, denosumab, anticonvulsant) at the time of the calcium load test.

**Figure 2.**
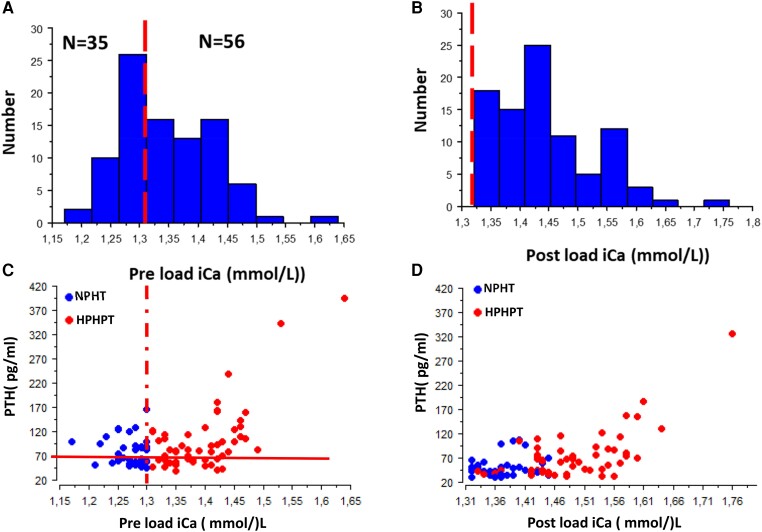
Distribution of iCa in the studied population in a fasting state after (A) a calcium-restricted diet and (B) following a calcium load test. As shown, all patients experienced a postload iCa >1.31 mmol/L. Relationship between PTH and iCa (C) before and (D) after a calcium load test. Dotted lines represent the iCa normal upper limit. In (C) the solid line represents the serum PTH normal upper limit. In (D) the solid line represents serum the PTH upper limit in the context of hypercalcemia (see “Material and Methods”).

Among the 91 patients with hyperparathyroidism, we were able to identify 2 groups: a normocalcemic group NHPT (n = 35 patients) with a normal fasting (preload) serum ionized calcium level (iCa < 1.31 mmol/L) and a hypercalcemic group HPHPT (n = 56 patients) with a high fasting serum ionized calcium level. Figure 2A and 2B shows the distribution of preload and postload iCa values.

A total of 58 patients, 51% with NHPT (n = 18) vs 71% of HPHPT (n = 40), underwent parathyroid resection (*P* = .16), with 1 parathyroid gland removed in most, except 5 patients with HPHPT in whom 2 glands were removed.

Gland weight values were collected from operative reports of patients and when several glands were removed, the sum of the gland weights was calculated. The reports were retrieved from the archive files of patients from the Department of Physiology (Hôpital Tenon, APHP) and from the files of the Pathology Department (Hôpital Tenon, APHP).

Clinical and biological data were collected from the Department of Physiology database (data collection was approved by the “Commission Nationale de l’Informatique et des Libertés” according to French legislation, no. 2065902v0).

### Calcium Load Test

A 24-hour urine collection under a regular diet was performed at baseline to measure several parameters, including diuresis volume and calcium excretion. After a 2-day calcium-free diet including low-calcium drinking water (<10 mg/L of elemental calcium), patients were referred to our unit for an oral calcium load. Briefly, a fasting blood sample was analyzed for total and ionized calcium, phosphate, magnesium, creatinine, uric acid, bicarbonates, PTH, and 25(OH)-D3 and 1,25(OH)-D3 vitamins. Bone remodeling biomarkers (serum bone alkaline phosphatase) were assayed at that time. A fasting urine sample over a 30-minute period was also collected to measure calcium, creatinine, and phosphate. Phosphate tubular reabsorption rate and creatinine clearance were measured, and TmPO_4_/GFR was calculated according to the method of Bijvoet and Morgan (7). An oral calcium load (calcium carbonate 1 g by mouth) was given, and 90 minutes later another 2-hour urinary collection was performed (analyzed for calcium, creatinine, and phosphate) with simultaneous blood sample analysis for total and ionized calcium, phosphate, creatinine, and PTH (6). All of the patients were subjected to a dietary inquiry to evaluate intakes over 1 week on their usual diet, including calcium intake.

### Assays

Total plasma and urinary calcium and magnesium were determined by atomic absorption spectrometry (PerkinElmer 3300). Ionized calcium, total CO_2_, sodium, and potassium were analyzed using a specific electrode (ABL 815, radiometer), urinary pH using a specific electrode, phosphate using a standard colorimetric method, plasma and urinary creatinine by the enzymatic method using the Konelab 20 analyzer (Thermo Fisher Scientific), PTH, 25 OH vitamin D, and 1.25 OH vitamin D were measured using a radioimmunological assay kit (immunodiagnostic and international Cisbio system). Plasma markers of bone remodeling (tartrate-resistant acid phosphatase and bone alkaline phosphatase were assessed using an enzymatic immunodiagnostic assay (Cisbio immunodiagnostic and international system). Terminus FGF23 C was measured using an immunotropic enzyme-linked immunosorbent assay.

A total of 60 stones were collected and analyzed by both morphology and infrared, enabling the main component to be identified.

### Estimation of Various Biological Variables

The glomerular filtration rate expressed in mL/min/1.73 m^2^ was estimated by the CKD EPI formula (8). Calcium gut absorption was assessed by the difference between preload and postload urinary calcium/creatinine ratio expressed in mmol/mmol creatinine. Fractional excretion of calcium (FECa) was calculated according to the following formula: (U Ca [mmol/L] × (P creatinine [mmol/L])/(iCa [mmol/L] × U creatinine [mmol/L]) (U, urinary; P, plasma). Preload and postload FECa variation (expressed in %) was calculated using the following formula: (FECa postload − FECa preload) ×100/(FECa preload). Preload and postload PTH variation (expressed in %) was calculated as follow: (PTH postload − PTH preload) × 100/PTH preload.

### Statistical Analysis

Qualitative and quantitative data were reported as percentage and mean ± SD or median with interquartile interval, respectively. The comparison of quantitative and qualitative data were carried out according to the Mann–Whitney and chi-squared tests, respectively. A *P* value strictly less than .05 was considered to be statistically significant. All statistical analyses were carried out using Statview 5.0 software.

## Results

As shown in Fig. 1, among a renal stone population of 1671 patients included retrospectively, 35 patients had normal preload ionized calcium (iCa ranging from 1.17 to 1.31 mmol/L) while 56 had hypercalcemia following a 48-hour calcium-restricted diet. All patients were hypercalcemic following calcium load (Fig. 2) with the ionized calcium level ranging from 1.31 to 1.75 mmol/L and serum PTH level ranging from 39 to 394 pg/mL. In NHPT, 51% of patients (18/35) had a fasting iCa and serum PTH concentrations within the normal range (Fig. 2C). According to preload total serum calcium values, 4/35 patients with NHPT would have been classified as HPHPT, and PHPT would have been misdiagnosed in 12/35 patients with NHPT who experienced a normal postload total serum calcium. Conversely, 7/56 and 54/56 patients with HPHPT would have been misclassified respectively as NHPT and “normal” according to preload and postload total serum calcium values. Median age is 56.4 years with a sex ratio of 28/63 for males and females respectively and a normal eGFR (Table 1). The major component of kidney stones in our population is calcium oxalate dihydrate in 32.5% of cases, mixed stones of calcium oxalate dihydrate and calcium oxalate monohydrate in 26.9% of cases, and calcium oxalate monohydrate as the major component in 9.6% of cases. Calcium phosphate as the major component is present in 31% of stones, with carbapatite in 25% of cases and brushite in 6% of cases. To sum up, 90.4% of stones are related to hypercalciuria with no difference between NHPT and HPHPT groups (*P* = .87).

**Table 1.** Comparison of biological data between NHPT and HPHPT groups

	N	Whole population	NHPT	HPHPT	*P*
Calcium diet (mg/day)	91	516 (286-696)	527 (284-696)	466 (305-689)	.1
Parathyroid gland weight (mg)	45	400 (200-1045)	273 (127-700)	500 (260-1250)	.05
Serum creatinine (µmol/L)	91	66 (55-79)	60 (51-80)	67 (58-77)	.42
eGFR*^a^* (mL/min/1.73 m^2^)	91	95.5 (86-106)	95.5 (83-105)	95.4 (87-107)	.32
Preload iCa (1.14 < N < 1.31 mmol/L)	91	1.33 (1.29-1.41)	1.28 (1.25-1.29)	1.38 (1.35-1.43)	<.0001
Postload iCa (1.14 < N < 1.31 mmol/L)	91	1.43 (1.37-1.49)	1.37 (1.34-1.40)	1.48 (1.43-1.54)	<.0001
Preload calcemia (2.20 < N < 2.51 mmol/L)	91	2.58 (2.47-2.67)	2.46 (2.37-2.51)	2.63 (2.57-2.70)	<.0001
Postload calcemia (2.20 < N < 2.51 mmol/L)	91	2.75 (2.63-2.85)	2.61 (2.48-2.73)	2.80 (2.72-2.92)	<.0001
Fasting phosphatemia (0.85 < N < 1.31 mmol/L)	91	0.78 (0.67-0.85)	0.80 (0.69-0.85)	0.77 (0.65-0.85)	.53
CO_2_t (23 < N < 27 mmol/L)	91	25.5 (23.7-27.2)	26.8 (24.9-28.6)	27.4 (25.2-28.8)	.45
Preload PTH (12 < N < 75 ng/mL)	91	80 (58-109)	66 (55-94)	82 (61-113)	.13
Postload PTH (12 < N < 75 ng/mL)	91	51 (40-76)	43 (36-55)	64 (43-85)	.003
Delta PTH (%)	91	−31.8 (18-44)	−36.7 (24-50)	−28 (28-42)	.02
Magnesemia (0.75 < N < 1.20 mmol/L)	91	0.84 (0.80-0.89)	0.85 (0.8-0.89)	0.84 (0.79-0.89)	.9
25OH vitamin D (30 < N < 70 ng/mL)	91	24 (17-30)	26 (23-37)	21 (12-25)	.04
1,25(OH)_2_ vitamin D (17 < N < 67 pg/mL)	91	77 (60-96)	77 (60-96)	80 (62-101.5)	.81
FGF23 (RU/mL)	91	71 (52-101)	77 (61-94)	69 (52-105)	.76
BALP (ng/mL)	91	15.7 (11.7-23.9)	15 (11-23)	17 (12-24)	.29
Preload TRP (N > 84%)	91	85 (81-89)	86 (81-90)	85 (80-88)	.26
Preload TmP/GFR (N > 0.77 mmol/L)	91	0.65 (0.57-0.73)	0.66 (0.59-0.76)	0.69 (0.57-0.79)	.36
Calciuria (mmol/day)	91	7.2 (4.8-11.6)	7.0 (4.9-11.2)	7.8 (4.7-12.7)	.75
Preload U Ca (mmol/mmol creatinine)	91	0.51 (0.26-0.64)	0.28 (0.21-0.60)	0.54 (0.32-0.68)	.007
Postload U Ca	91	1.3 (0.8-1.9)	1.2 (0.8-1.7)	1.3 (0.9-2.0)	.35
Delta U Ca (mmol/mmol creatinine)*^a^*	91	0.8 (0.5-1.2)	0.8 (0.5-1.2)	0.8 (0.5-1.2)	.92
Fasting U pH	91	6.3 (5.7-6.6)	6.3 (5.9-6.7)	6.3 (5.7-6.6)	.44
Fasting U FECa (%)	91	2.3 (1.3-3.0)	1.7 (1.1-2.6)	2.5 (1.6-3.5)	.003
Post load U FECa (%)	91	5.9 (3.9-8.1)	5.3 (3.7-8.0)	6.0 (4.2-8.1)	.74
U FeCa ratio (%)*^b^*	91	177 (104-267)	207 (137-402)	171 (92-222)	.01

Abbreviations: BALP, bone alkaline phosphatase; CO_2_t, plasma bicarbonate; eGFR, estimated glomerular filtration rate using the CKD epi formula; FECa, fractional excretion of calcium (expressed in %); TmP/GFR, renal threshold phosphate concentration; TRP, tubular reabsorption of phosphate; U, urinary.

^*a*^Difference between postload and preload urine calcium (expressed in mmol/mmol creatinine);

^*b*^Difference between postload and preload U FECa/preload U FECa expressed in %.

**Figure 3.**
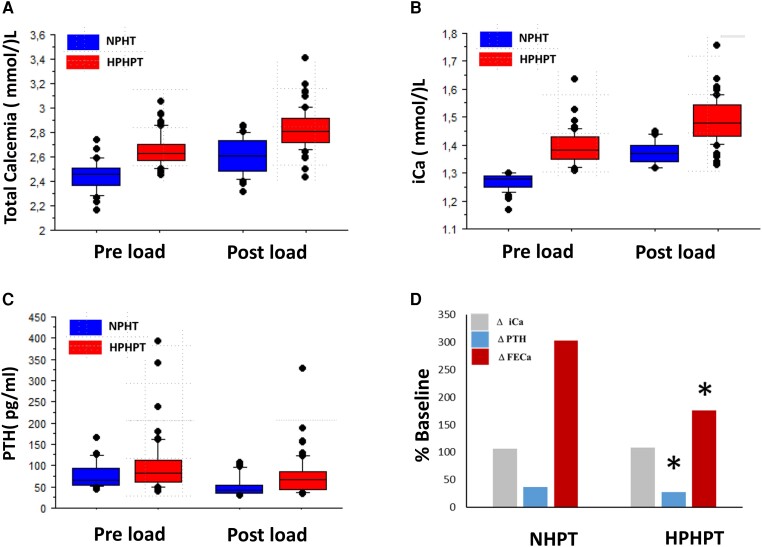
Comparison between NHPT and HPHPT groups of preload and postload total calcemia (A), iCA (B), PTH (C), and postload iCa, PTH FECa variation (D) (*P < 0.05). Results are expressed in percentage from baseline value (fasting value = 100%).

Despite significantly different preload ionized calcium values between NHPT and HPHPT groups, the preload PTH concentration and usual calcium diet assessed by the GRIO survey (9) were similar between NHPT and HPHPT groups (Table 1). However, after calcium load, PTH serum levels were higher in the HPHPT group (43 vs 64 pg/mL, *P* < .003) with lower PTH inhibition compared with NHPT (28% vs 37% respectively as shown Table 1 and Fig. 3). Serum calcitriol concentration was similar between the 2 groups despite higher 25-hydroxy-vitamin D concentration in NHPT (*P* = .04). The serum phosphate level is low in 70% and 67% of cases in HPHPT and NHPT respectively (*P* = NS) with no difference for TmP/GFR and FGF23 values.

**Figure 4.**
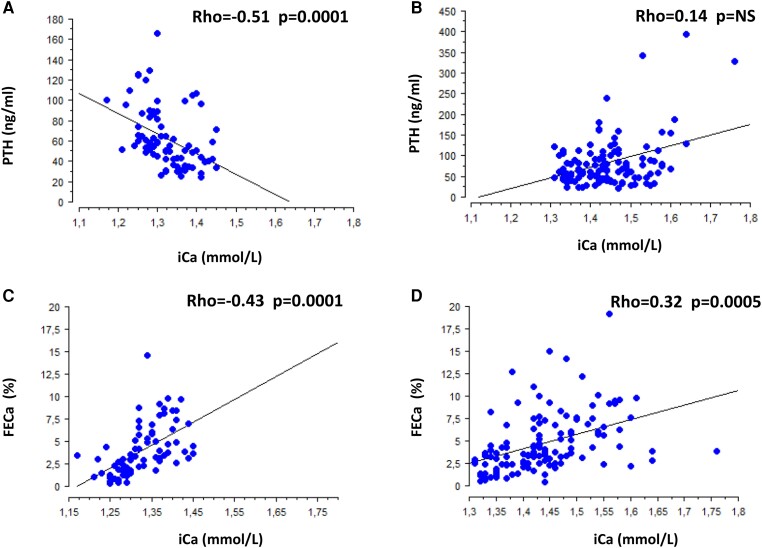
Association between iCa and serum PTH in NHPT (A) and HPHPT groups (B). Association between iCa and FECa in NHPT (C) and HPHPT groups (D). Patients’ preload and postload data are pooled.

Calcium absorption, assessed both by 24-hour calciuria on a free diet and by urinary calcium/creatinine ratio increase (delta UCa) before and after calcium load, was similar in the 2 groups (Table 1). Though fasting renal calcium excretion assessed by FECa is higher in the HPHPT group (2.3% vs 1.7% *P* = .003), FECa after calcium load was similar between the 2 groups despite a higher iCa. Indeed, as shown Fig. 3, after calcium load, urinary calcium excretion increased significantly less in the HPHPT group than in the NHPT group (mean value 176% vs 303%, *P* = .01) according to lower PTH inhibition in the HPHPT group. Accordingly, as shown Fig. 4, when pooling a patient's preload and postload data, a strong negative association was detected between iCa and PTH in the NHPT group whereas no association was present in the HPHPT group (Fig. 4A and 4B, respectively) and the slope of the association between iCa and FECa was steeper in the NHPT than the HPHPT group (Fig. 4C and 4D, respectively).

Eighteen patients with NHPT (51%) and 40 patients with HPHPT (71%) underwent parathyroidectomy (*P* = NS) (Fig. 1). Parathyroid gland weights were available in 40 cases with a median value of 870 mg and surprisingly, despite a trend, no significant difference (*P* = .05) was observed between the NHPT and the HPHPT groups (294 and 500 mg respectively as shown in Table 1) with 22% vs 35% of glands above 1000 mg in the NHPT and the HPHPT group, respectively (*P* = NS).

No association was found between iCa and gland weight with iCa values of 1.34 ± 0.06 mmol/L vs 1.35 ± 0.06 mmol/L in the first and last quartile, respectively (*P* = NS).

Among the 18 patients with NHPT who underwent surgery, 8 had normal baseline PTH and iCa and 10 had normal baseline iCa and high PTH with a median PTH weight of 286 g and 400 g, respectively. Among this population, 11 underwent a new calcium load test following surgery and postload iCa was normalized in 9 cases.

## Discussion

NHPT is a debated entity and its definition varies among authors, explaining a reported prevalence which varies according to the studies of between 5.5% and 20% of patients with PHPT (3, 10-13). As recommended by the Fourth International Workshop on the diagnosis of asymptomatic PHPT (1, 14, 15), ionized calcium and not total serum calcium should be considered as the biological parameter defining hypercalcemia. Indeed, total calcium is a poor estimate of ionized calcium (16-18), which is indeed the regulated physiological value. Therefore, some so-called patients with normocalcemia are actually hypercalcemic when referring to the serum ionized calcium value (19). In our series, performed under a restricted calcium diet (calcium diet information is often a missing criterion in the literature), total preload calcemia was significantly different; 4/35 patients with NHPT have an increased value (between 2.58 and 2.67 mmol/L). Thus, NPHT classification based upon total calcemia following a 2-day restricted diet would have led to a misclassification into HPHPT for at least 4 patients. As pointed out, 11/56 patients with HPHPT would have been misclassified as NHPT based upon total serum calcemia, pointing to the lack of precision in total serum calcium measurements. True prevalence of NHPT is also dependent on the studied population (ie, patients with bone disease or renal stones) and requires quick processing of samples in laboratories, accurate measurements, and trained practitioners, as the assessment of NHPT relies on an inadequately high PTH level in patients with iCa within the normal upper limit values.

Our data show that in our series, in addition to 56 HPHPT cases, the diagnosis of NHPT was assessed in 35 patients with normal iCa and a high serum PTH in 49% of cases only. Indeed, a striking normal fasting iCa and serum PTH concentration was encountered in 51% of cases of NHPT, which would have been misdiagnosed without a calcium load test unmasking true hypercalcemia with an inadequate PTH concentration (within normal range but not low as one should expect in a normal counter-regulation), thus ruling out secondary hyperparathyroidism. Calcium intakes have an influence on calcemia and PTH measurements (20, 21). We speculate that among patients with NHPT, prevalence of fasting normal iCa may vary with calcium diet in some patients with normocalcemia under a 48-hour calcium restriction diet becoming hypercalcemic under a normal or a high-calcium diet, thus enabling a diagnosis of HPHPT without conducting a calcium load test. Fifty-one percent and 71% of patients with NHPT and HPHPT, respectively, underwent parathyroid surgery due to renal stone disease activity and/or severe hypercalcemia in some cases. No difference in stone composition between the 2 groups was found, with calcium-dependent stones related to hypercalciuria in 90% of cases, in other words, calcium oxalate dihydrate as the major compound in a majority of cases and calcium phosphate stones (brushite and/or carbapatite) in 30% of cases. No difference in patient age, gland weight, stone risk factors, or bone remodeling biomarkers was detected, suggesting that NHPT is not an early step in HPHPT or a mild form of PHPT, and thus raises the issue of the mechanism at play accounting for a fasting normocalcemia.

Indeed, our data show that on a regular diet, calcium intake and 24-hour calciuria are similar in the 2 groups, suggesting similar calcium intestinal absorption. As expected, fasting FECa is higher in patients with HPHPT given the higher glomerular calcium filtration load, but it plateaus, increasing only by 176% after calcium load compared with 303% in the NHPT group. Indeed, renal calcium excretion is more efficient in the NHPT group: for similar iCa values, FECa was 5.3% in NHPT (postload value with iCa:1.37 mmol/L) and only 2.5% in HPHPT (fasting value with iCa 1.38 mmol/L). A lower postload serum PTH concentration (with a 36% decrease compared with 28% in the HPHPT group) may explain this finding and thus points out more severe parathyroid dysfunction in HPHPT than NHPT, although gland weights were similar in the 2 groups.

Among the 18 patients with NHPT who underwent surgery, we performed a postoperative load test for 11 patients. Nine out of 11 patients had a normal calcium load test and 2 had postload hypercalcemia (postload PTH <30 pg/mL in 1). These 2 patients had elevated preload PTH before surgery.

The finding that pooled preload and postload iCa were strongly negatively associated with serum PTH only in the NHPT and not the HPHPT group suggests preserved CaSR signaling within parathyroid cells following proliferation into adenoma or hyperplasia, conversely to patients with HPHPT where a significant contingent of parathyroid cells more likely underwent CaSR downregulation disturbing normal calcium homeostasis. Zavatta also hypothesized the role of CaSR in NHPT physiopathology (22). This speculation should deserve further confirmation through specific histological studies.

### Limitation of the Study

This observational retrospective study allows, through extensive biological phenotyping, an accurate diagnosis of NHPT even when fasting iCa and serum PTH within normal values may be questionable and could suggest inaccurate fasting iCa or serum PTH measurements, as PHPT diagnosis was confirmed in all patients. Our results suggest that though the time lag between blood sample and measurement was less than 15 minutes and serum PTH measurement was performed using a robust radioimmunoassay, normal iCa and serum PTH measurements do not rule out NHPT with 100% specificity.

Four patients (11%) classified in the NHPT group were hypercalcemic according to total serum calcium, whereas 7 (12.5%) were misclassified in the HPHPT group. Thus, according to total serum, the NHPT and HPHPT groups would be slightly different, with 38 and 53 patients, respectively.

As imaging was performed only when surgery was considered depending on a patient's intention, biology, and, especially in the NHPT group, magnitude of hypercalciuria under a controlled diet and/or bone density, we cannot provide data on how many patients had a visible target on ultrasound or dynamic imaging.

Conversely to what was expected, in our series, no patients with NHPT had multiple gland disease, whereas 5 patients with HPHPT had 2 parathyroid glands removed. This discrepancy may be due to the criteria selected to define NHPT as mentioned above: calcium restricted diet or not, total serum calcium vs iCa, delay between processing samples, and last the performance of a calcium load test to assess the diagnosis.

Our study also does not allow any conclusion about follow-up of patients with NHPT as the monitoring of the 17 patients with no surgery is ongoing and we do not know how many will become HPHPT in the future and/or will undergo a parathyroid surgery. The indication of surgery especially in NHPT is indeed a matter of debate: though surgery decreases significantly stone recurrence (23) and exerts a beneficial effect on cardiovascular risk factors (24), persistent hypercalciuria may indeed remain to a lesser degree in some cases. Alternatively, a conservative attitude in the absence of a surgical target, or even when a surgical target is found (25), with adequate monitoring and appropriate intake of calcium and vitamin D seems, according to our findings, debatable in patients with NHPT with active hypercalciuric stone disease.

## Conclusion

To conclude, this study demonstrates that 40% of patients with a final diagnosis of PHPT are normocalcemic following a 48-hour calcium-restricted diet, with half of NHPT having apparently normal calcium homeostasis despite hypercalciuria (ie, normal fasting iCa with normal serum PTH concentration) while the other half meet the expected criteria defined by “a normal total and ionized serum calcium level without any other known etiology for a secondary elevation of parathyroid hormone (PTH)” (2, 3). Our data thus pinpoint potential undiagnosed patients with NHPT in the hypercalciuric renal stone population. We suggest performing either nonfasting iCa and PTH measurements in patients under a regular diet or alternatively a calcium load test whenever necessary, in particular in normocalcemic hypercalciuric patients with active renal stone disease under a controlled diet.

## Disclosures

The authors have nothing to disclose.

## Data Availability

Original data generated and analyzed during this study are included in this published article.

## Abbreviations

FECa: fractional excretion of calcium
HPHPT: hypercalcemic hyperparathyroidism
iCA: ionized calcium
NHPT: normocalcemic primary hyperparathyroidism
PHPT: primary hyperparathyroidism
PTH: parathyroid hormone
